# Radioactive iodine-refractory differentiated thyroid cancer: an uncommon but challenging situation

**DOI:** 10.1590/2359-3997000000245

**Published:** 2017-01-27

**Authors:** Angelica Schmidt, Laura Iglesias, Michele Klain, Fabián Pitoia, Martin J. Schlumberger

**Affiliations:** 1 Hospital de Clínicas University of Buenos Aires Buenos Aires Argentina Division of Endocrinology, Hospital de Clínicas, University of Buenos Aires Buenos Aires, Argentina; 2 Institut Gustave Roussy Université Paris-Saclay Villejuif France Institut Gustave Roussy, Université Paris-Saclay, Villejuif, France; 3 Università Federico II di Napoli Napoli Italia Università Federico II di Napoli, Napoli, Italia

**Keywords:** Differentiated thyroid cancer, radioactive iodine refractory thyroid cancer, tyrosine kinase inhibitors

## Abstract

Radioiodine (RAI)-refractory thyroid cancer is an uncommon entity, occurring with an estimated incidence of 4-5 cases/year/million people. RAI refractoriness is more frequent in older patients, in those with large metastases, in poorly differentiated thyroid cancer, and in those tumors with high 18-fluordeoxyglucose uptake on PET/CT. These patients have a 10-year survival rate of less than 10%. In recent years, new therapeutic agents with molecular targets have become available, with multikinase inhibitors (MKIs) being the most investigated drugs. Two of these compounds, sorafenib and lenvatinib, have shown significant objective response rates and have significantly improved the progression-free survival in the two largest published prospective trials on MKI use. However, no overall survival benefit has been achieved yet. This is probably related to the crossover that occurs in most patients who progress on placebo treatment to the open treatment of these studies. In consequence, the challenge is to correctly identify which patients will benefit from these treatments. It is also crucial to understand the appropriate timing to initiate MKI treatment and when to stop it. The purpose of this article is to define RAI refractoriness, to summarize which therapies are available for this condition, and to review how to select patients who are suitable for them.

## INTRODUCTION

Despite the high and increasing incidence of differentiated thyroid carcinomas (DTCs), only a few patients (less than 10% of patients with clinical disease) will develop distant metastases. Two thirds of these patients will become refractory to the treatment with radioactive iodine (RAI), and they represent 4-5 new cases/year/million. After the discovery of advanced RAI-refractory disease, the 10-year survival rate is usually less than 10% and the mean life expectancy is 3-5 years ( 1 ). Systemic chemotherapy has limited efficacy with a high toxicity rate ( 2 ). Multikinase inhibitors (MKIs) are the most investigated drugs. Two of these compounds, sorafenib and lenvatinib, have shown objective response rates and have significantly improved the progression-free survival rates in the two largest published prospective randomized trials performed with an MKI in patients with advanced refractory DTC. However, no overall survival benefit has been demonstrated yet. This is probably related to the crossover that occurs in most patients who progress on placebo treatment to the open treatment ( 3 , 4 ).

The aim of this review is to define RAI refractoriness and summarize the therapies currently available. We also aim to analyze the most appropriate timings to initiate and to stop MKI treatment.

### Defining RAI refractoriness

RAI treatment is the first-line systemic treatment in patients with advanced disease ( 5 ). Achieving a cure with RAI treatments is frequent in young patients with small metastases from well-differentiated thyroid cancer who have high uptake of RAI in neoplastic foci. These patients represent about one third of all patients with an advanced form of the disease. Partial response and long-term stabilization may be obtained, but cure is rarely achieved in the other two thirds of patients with an advanced form of the disease, who will be classified as refractory at some point during their life ( 1 , 6 ).

It is important to recognize at which point RAI treatment is no longer beneficial for DTC patients in order to avoid unnecessary treatments that may lead to severe adverse events (AEs) and to consider alternative local or systemic therapies ( 5 , 7 ). Indeed, the practitioner should ascertain that decreased RAI uptake is not due to iodine contamination or to insufficient TSH (thyrotropin) stimulation ( 8 ). When this has been excluded, there are different possible scenarios ( 5 , 6 ):

#### Metastatic disease that does not take up radioactive iodine at the time of the first I131 treatment

For these patients, treatment with I^131^ does not provide any benefit. This group includes patients with structurally evident disease with no RAI uptake on a diagnostic, whole-body scan. In some of these patients, RAI uptake may be observed on post therapy scans but usually will not be high enough to induce any therapeutic benefit ( 9 ).

#### Ability to take up RAI lost after previous evidence of uptake

This is frequently observed in patients with multiple large metastases, and it is generally due to the eradication of differentiated tumor cells with RAI uptake, with persistence of those poorly differentiated clones that will continue growing ( 6 ).

#### RAI uptake retained in some lesions but not in others

This situation is also frequently seen in patients with multiple large metastases, and progression is likely to occur in metastases without RAI uptake, in particular when high 18-fluorodeoxyglucose (^18^FDG) uptake is present ( 10 , 11 ).

#### Metastatic disease that progresses despite substantial uptake of RAI

When structural progression occurs within 12 to 16 months after the course of an adequate RAI treatment, subsequent RAI treatment, even with higher activities, will be ineffective ( 12 ).

#### Absence of complete response to treatment after > 600 mCi of cumulative activity of RAI

The situation is less clear in patients who still have visible RAI uptake in all lesions and who are not cured, despite several treatment courses, but in whom disease does not progress according to Response Evaluation Criteria In Solid Tumors (RECIST) 1.1 criteria ( 13 ). The probability of obtaining a cure with further RAI treatment is low, and the risk of AEs increases with further treatments ( 1 , 5 , 7 ). The decision to continue RAI treatment is generally based on the magnitude of tumor response to previous treatment courses, the persistence of a significant RAI uptake, a low ^18^FDG uptake in tumor foci, and the absence of detectable side effects ( 6 ).

#### High uptake of 18FDG on PET/CT scan

The likelihood of obtaining a complete response is reduced when ^18^FDG uptake on PET/CT scanning is high in the tumor foci. However, the decision to abandon RAI therapy should not be based only on the presence or intensity of ^18^FDG uptake ( 14 - 17 ).

#### Advanced disease and unfeasible thyroidectomy

When the thyroid gland has not been removed, RAI treatment is usually not administered and RAI uptake status cannot be assessed. These patients are usually managed as iodine-refractory patients ( 6 ).

## Rationale for the use of MKIs

Genetic alterations inducing the activation of the *RAS-RAF-MEK-ERK* and *PI3K/Akt/mTOR* signaling pathways are found in the majority of DTCs ( 18 ). Angiogenic factors are also involved in the cellular control of differentiation, proliferation, and survival. Vascular endothelial growth factor (VEGF) stimulates endothelial cell proliferation and is a key to tumor angiogenesis. VEGF has an important role in thyroid cancer development, and its expression level correlates with advanced disease ( 19 , 20 ). As a consequence, MKIs targeting angiogenesis have recently been used with encouraging results in clinical trials involving patients with progressive and unresectable RAI-refractory disease ( 3 , 4 , 21 ).

### When to initiate an MKI?

One main challenge is properly selecting patients for systemic therapy. As all these medications can cause a decrease in quality of life (QoL) and life-threatening, adverse effects, it is important to identify which patients may benefit from and should be placed on therapy. Patients with distant metastases may have a disease that does not progress for years. In these patients, it is recommended to keep TSH suppression therapy with levothyroxine and imaging every 3-12 months (CT scan, ^18^FDG-PET/CT scan, or MRI) based on the disease burden and location of lesions ( 5 , 22 ). Although serum thyroglobulin (Tg) levels are measured as a biomarker of the disease extent, patients should not be identified as having progressive disease only on the basis of rising levels of serum Tg. Rapidly increasing serum Tg levels should, however, lead to more frequent and comprehensive imaging in efforts to identify structural correlates ( 23 ).

In general, the appropriate indications to initiate a MKI treatment are ( 5 , 24 , 25 ) as follow:

### Rapidly progressive disease and large tumor burden

Large, multiple tumors greater than 1-2 cm in size that are rapidly progressing (within < 12 months) should be considered for treatment; in these patients, treatment should preferably be initiated before the occurrence of symptoms ( 6 , 26 ).

In contrast, patients with smaller tumors (< 1 cm) or with only a few lesions and with no documented progression rarely require immediate, systemic treatment with an MKI ( 6 ).

For patients with smaller tumors that are rapidly progressing (< 6-12 months) or for those who have large tumors that progress slowly ( > 12 months), the decision to treat or not (or to postpone treatment) is less clear and should be considered on a case-by-case basis ( 6 , 26 , 27 ).

### Symptomatic disease and the risk of local complications

Dyspnea or painful bone lesions should first be submitted to focal therapy. Also, symptomatic treatment modalities are always warranted, as well as bisphosphonates or an anti-RANK ligand antibody in patients with bone metastases. Cases of ineffectiveness or with the presence of tumor foci near the respiratory–digestive axis or large vessels may be an indication to initiate treatment, even in patients with no demonstrated progression before the occurrence of tumor involvement of the trachea or esophagus and before encasement of great vessels that may contraindicate the use of an MKI with respect to the risk of bleeding ( 28 ).

### Good overall performance status and acceptable life expectancy

Before initiation, a comprehensive review is necessary to ascertain the patient’s suitability for therapy. An initial evaluation includes assessment of the patient’s performance status. Little is known about the tolerability of MKIs in patients with a poor performance status ( *e.g* ., ECOG 2 or more) because all trials with MKIs have excluded these patients ( 29 ).

### Absence of comorbidities or contraindications

Cardiovascular history, poor blood pressure control, and hematological, renal, and hepatic abnormalities may contraindicate any MKI treatment or may indicate treatment initiation at a lower dosage ( Table 1 ) ( 5 , 28 ).

**Table 1:** Contraindications or factors discouraging MKI treatment

Contraindications	Comment
Intestinal or liver disease	Active or recent diverticulitis, inflammatory bowel disease, or recent bowel resection Laboratory: AST-ALT > 5 times the upper limit of normal range; increased bilirubin level
High risk of bleeding	Recent gastrointestinal hemorrhage or hemoptysis, coagulopathy, or anticoagulant treatment Tumor involvement of the larynx, trachea–bronchus axis or the pharyngo-esophagus axis Encasement of great vessels
High cardiovascular risk	Unstable angina, myocardial infarction, or stroke within 6 months prior to MKI initiation
Poorly controlled hypertension	Uncontrolled hypertension; start antihypertensive treatment first if blood pressure is > 140/90 mmHg
Prolonged QTc interval	≥ 450 msec History of ventricular arrhythmias and bradyarrhythmias
Renal impairment	CrCl < 60 ml/min Proteinuria ≥ 1g/24h
Recent tracheal radiation therapy	Within 6 months prior to MKI initiation Increased risk of bleeding/fistula
Cachexia, poor nutrition, sarcopenia	Care should improve performance status
Untreated brain metastases	Controversial
Recent suicidal ideation	Suicide has been reported in depressed patients receiving MKIs
Concomitant medication that induces or inhibits CYP3A4	Avoid or substitute for another drug. If a CYP3A4-inhibiting drug cannot be eliminated, consider a dose reduction in the MKI
Life expectancy	If it is too brief systemic therapy will not be justified

ALT: alanine aminotransferase; AST: aspartate aminotransferase; CrCl: creatinine clearance.

### Good compliance to treatment

Due to the duration of treatment, the potential for toxicities, and the need for regular monitoring, patients must be aware that the follow-up will be close and may be prolonged for years.

## Which agents are available?

### Multikinase inhibitors

*Sorafenib*

Sorafenib targets *BRAF, RET, VEGFR 1–3, PDGFR,* and *c-KIT* and was the first agent approved (in 2013) for the treatment of refractory DTC, based on the DECISION trial, a randomized, placebo-controlled, phase III trial ( 30 , 31 ). A total of 417 adult patients with progressive advanced RAI-refractory DTC were randomized 1 to 1 to sorafenib (800 mg daily) or a placebo. The median progression-free survival (PFS) of patients treated with sorafenib was significantly improved compared to the placebo (10.8 vs. 5.8 months; p < 0.0001). Disease control rate, including partial responses (PRs in 12% of patients) and stable disease (SD > 23 weeks), was achieved in 54% of patients treated with sorafenib. There was no difference in overall survival (OS), even after correction of the potential benefits of the crossover in patients from the placebo group who crossed over to the sorafenib treatment upon disease progression. AEs occurred in almost all patients, but most were grade 1 or 2. The most frequent AEs were dermatological – hand–foot syndrome (76%), alopecia (67%), and rash or desquamation (50%) – but also included diarrhea (68%), fatigue (49%), weight loss (46%), and hypertension (40%). Serious AEs occurred in more than 30% of patients, the most frequent being secondary malignancy (4.3%), dyspnea (3.4%), and pleural effusion (2.9%). The dose was decreased in 64% of cases, and the drug was discontinued due to AEs in 19% of patients ( 32 ).

*Lenvatinib*

Lenvatinib targets *VEGFR1-3, FGFR1-4, PDGFR-b, RET* , and *c-KIT* and was labeled in 2014 based on the SELECT trial ( 33 ). SELECT, a phase III trial, enrolled a total of 392 patients with progressive, advanced RAI-refractory DTC who were randomized 2 to 1 to lenvatinib (24 mg/day) (n = 261) or a placebo (n = 131). The median PFS was significantly improved compared to the placebo (18.3 months vs. 3.6 months; p < 0.001). The PFS benefit was found among all subgroups, including patients previously treated with another MKI (25% of patients), distinct histology subtypes (i.e., papillary, poorly differentiated, follicular, and oncocytic), and site of metastases, and was independent of the *BRAF* and *RAS* mutational status of the tumor. In addition, a significant objective response rate of 64.8% was documented among patients treated with lenvatinib, including complete responses in 4 patients; furthermore, a prolonged stable disease (longer than 23 weeks) was observed in 15% of patients. Responses occurred rapidly after initiation of treatment, with a median time to response of only two months. Grade 3 or higher AEs occurred in 75% of patients and led to dose reductions in 67% and discontinuation of treatment in 14% of patients. The most frequent grade 3 or higher treatment-related AEs were hypertension (42%), proteinuria (10%), arterial and venous thromboembolic events (2.7% and 3.8%, respectively), acute renal failure (1.9%), QTc prolongation (1.5%), and hepatic failure (0.4%). Six deaths in the lenvatinib group were considered probably treatment related by the investigators: 3 cases resulted from unspecified causes and 3 were associated with pulmonary embolism, hemorrhagic stroke, and health deterioration. No significant OS benefit was demonstrated with lenvatinib, but after correction for the potential benefits of crossover in patients of the placebo group who were treated with lenvatinib upon progression with a prespecified method, the benefit in terms of OS became significant ( 3 ).

Direct comparison of these two treatments has not been performed, but lenvatinib seems to be more effective than sorafenib, both in improving PFS and in obtaining an objective tumor response ( Table 2 ). These data are even more meaningful when considering that the SELECT trial enrolled patients with more advanced and more aggressive disease (as shown by a shorter median PFS in the placebo arm), some of whom had been previously treated with an MKI.

**Table 2:** Phase III trials

	Sorafenib vs. placebo Decision	Lenvatinib vs. placebo Select
Patients (n)	207 vs. 210	261 vs. 131
CR	0% vs. 0%	1.5% (n = 4) vs. 0%
PR	12.2% vs. 0.5%	63.2% vs. 1.5%
SD > 23 weeks	41.8% vs. 33.2%	15.3% vs. 29.8%
PFS months (median)	10.8 vs. 5.8 (HR 0.59 p < 0.0001)	18.3 vs. 3.6 (HR 0.21 p < 0.001)
Grade 3-4 AEs	37.2% vs. 26.3%	75.9% vs. 9.9%
OS	NS*	NS*

CR: complete response; PR: partial response; SD: stable disease; PFS: progression free survival; HR: hazard ratio; AEs: adverse events; OS: overall survival; NS: not statistically significant.

* Probably related to the crossover that occurs in most patients who progressed from the placebo treatment to the therapy treatment.

*Pazopanib*

Pazopanib targets *VEGFR1-3, PDGFR-a* and *-b* , and *c-KIT.* In a phase II trial on involving 37 patients with previously treated advanced RAI-refractory thyroid cancer, a PR occurred in 49% of patients and SD occurred in 47% of patients. AEs included fatigue, hair and skin hypopigmentation, alopecia, diarrhea, nausea, vomiting, anorexia, weight loss, hypertension, elevated liver function tests, proteinuria, and hematologic cytopenias. Serious AEs were uncommon but included lower gastrointestinal hemorrhage (grade 3) and intracranial hemorrhage (grade 4). A dose reduction due to AEs was required in 43% of patients. Two deaths were potentially related to the drug. It is important to note that the patients included in this trial were allowed to be treated with up to two previous systemic treatment lines, and radiographic progression of the disease was requested in the 6 months preceding enrolment. These criteria led to the selection of highly aggressive RAI-refractory DTC patients ( 34 ).

*Cabozantinib*

Cabozantinib targets *c-MET, VEGFR2,* and *RET* kinases and is currently approved for the treatment of advanced medullary thyroid cancer. Among 15 patients with RAI-refractory DTC, a PR was achieved in 8 (53%). All patients experienced at least one AE, and nearly all were grade 3 or higher. The most common AEs were diarrhea, nausea, fatigue, and decreased appetite ( 35 ).

*Sunitinib*

Sunitinib targets *VEGFR, PDGFR, c-KIT, FLT3* , and *RET.* In a phase II trial of 28 patients with advanced RAI-refractory DTC, sunitinib induced a PR in 28%, a CR in 1 patient, and SD in 46%. The most common AEs included fatigue, neutropenia, hand–foot syndrome, hypertension, and diarrhea. Four patients discontinued treatment due to toxicity; there were two serious bleeding episodes ( 36 ).

*Vandetanib*

Vandetanib targets *RET, VEGFR,* and *EGFR* . A randomized, double-blind, phase II trial enrolled 72 patients to the vandetanib group and 73 patients to the placebo group. Patients who received vandetanib had longer median PFS than those who received the placebo (11 months vs. 5.9, p < 0.05). PR and SD were observed in 8% and 57% of patients in the vandetanib arm, respectively. The incidence of grade 3 AEs was 53% in the vandetanib group. QTc prolongation and diarrhea were the most common AEs, and other frequent AEs included hypertension, rash, acne, and decreased appetite ( 37 ).

*Motesanib*

Motesanib targets *VEGF-R, PDGF-R,* and c- *KIT* . In a phase II study on 93 patients who had progressive, RAI-resistant DTC, PR was observed in 14% and SD longer than 23 weeks was observed in 35%. Nearly all patients (94%) had at least one AE, being grade 3 or more in half of them. The most commonly reported AEs were diarrhea, hypertension, fatigue, and weight loss ( 38 ).

*Axitinib*

Axitinib is an inhibitor of *VEGF-R 1-3, c-KIT,* and *PDGF-R.* In a phase II study on 52 patients with refractory DTC, a PR was observed in 38% and SD in 30%. Almost all patients experienced AEs, the most common grade 3-4 being hypertension, proteinuria, diarrhea, weight loss, and fatigue ( 39 , 40 ). Similar results were found in another phase II trial ( 41 ).

*Nintedanib*

Nintedanib targets *VEGF-R, FGF-R, PDGF-R* and *RET, Flt-3* , and *Src.* Based on promising efficacy and safety results in many other solid tumors, nintedanib is currently under investigation in DTC ( 42 , 26 ).

### Other treatment modalities

*Chemotherapy*

The most frequently used agent is doxorubicin. Phase II studies provided low and transient partial responses of 0 to 20%. Too few data exist to recommend other specific cytotoxic regimens, and their use within the context of a therapeutic clinical trial should be preferred ( 2 , 5 ).

*Inmunotherapy*

Some tumors evade immunosurveillance, which can occur through an inhibition of T-cell function induced by the expression of molecules such as CTLA-4, PD-1, or PD-L1 ( 43 , 44 ). Treatment with antibodies directed against checkpoint inhibitors ( *e.g* ., PD-1/PD-L1) has shown promise in other cancer types and is being investigated in advanced RAI-refractory thyroid cancer, used either alone or in combination with an MKI or an RAI ( 5 , 45 ).

*Selective BRAF inhibitors*

*Vemurafenib:* in a retrospective review of 15 patients with advanced PTC harboring the *BRAF* ^*V600E*^ mutation, a PR was observed in 47% ( 46 ).

*Dabrafenib* : among 14 patients, dabrafenib induced 4 (29%) PRs, and 64% of patients achieved at least a 10% reduction in tumor size ( 47 ).

*Crizotinib*

Crizotinib is an inhibitor of anaplastic lymphoma kinase ( *ALK* ) and *c-MET.* Recently, rearrangements involving the *ALK* gene were discovered in rare, poorly differentiated, and anaplastic thyroid cancers and, more frequently, in radiation-induced DTC ( 48 , 49 ). Crizotinib may be used in patients with a demonstrated *ALK- or c-MET* -activating mutation ( 50 ).

*Everolimus*

Everolimus is an inhibitor of *mTOR* . Activation of the PI3 kinase pathway occurs mostly in poorly differentiated thyroid cancers in addition to the activation of the MAP kinase pathway ( 51 ). In a phase II study on 38 patients with advanced thyroid cancer of any histology, only 2 (5%) patients achieved a PR. The AEs were predominantly grade 1 or 2, and the most common was mucositis (84%) ( 52 ). Another trial investigating everolimus’s combination with sorafenib is ongoing, and preliminary results have shown a synergistic effect, with PR in 58% of patients ( 33 ).

*Selumetinib*

Selumetinib is an MEK inhibitor that blocks the MAPK signaling pathway. It was used as a redifferentiating agent and increased the uptake of I^124^ in 12 (60%) of 20 patients with advanced refractory thyroid cancer; 8 of these 12 patients reached the dosimetry threshold for RAI therapy, and 5 of them achieved a PR after RAI treatment. It seems to be more effective in patients with *RAS* -mutated disease ( 53 ). Similar results were achieved in a phase II study with dabrafenib on 10 patients with *BRAF* ^*V600E*^ mutated thyroid cancers: 60% developed RAI uptake and 83% of patients showed a decrease in the size of target lesions after 6 months of RAI treatment, but only 2 patients met criteria for partial response ( 54 ).

### Combined therapies

Most patients eventually progress after responding to a first-line treatment, though some must discontinue the drugs due to toxicity. As a consequence, a number of studies have looked at sequencing MKI administration or the use of MKIs in the second-line setting for RAI-refractory DTC in patients whose cancers have progressed while receiving a first-line agent. These data suggest that a second-line TKI can be effective, with similar benefits in terms of PFS ( 55 , 56 ).

*Adverse events*

Education should be provided to each patient and to care providers. After initiation of treatment, it is highly recommended that clinicians follow-up with patients at 2-week intervals for the first 2-3 months and then once a month in order to proactively manage AEs.

The most common AEs and their management are presented in Table 3 ( 57 - 59 ). Less common but serious AEs are hypertension, arterial and venous thrombotic events, bleeding, gastrointestinal fistula and perforation, acute myocardial infarction, heart failure, secondary malignancies (squamous cell carcinoma), cytopenias, hepatotoxicity, renal failure, and reversible posterior leukoencephalopathy syndrome ( 3 , 4 ).

**Table 3:** Common adverse events of MKIs

	MKI most frequently involved (frequency of any grade of AE)	Management
Fatigue and loss of weight	All (26-59%)	Increase or at least maintain physical activity; take pills in the evening Monitor other causes (e.g., anemia, depression, electrolyte disturbance, hypothyroidism)
Diarrhea	All (30-68%)	Loperamide and/or codeine Dietary changes (eat low-fiber foods; avoid high-fat or spicy foods, alcohol, and caffeinated or carbonated drinks)
HTA	All (30-67%)	Monitor blood pressure at least once a week Diuretics, ACEIs, ARBs, BBs, or CCBs alone or in combination Avoid diltiazem, verapamile, and nifedipine
Rash	All (20-50%)	Use perfume-free soaps and wear loose, natural-fabric clothing; avoid hot or cold water Topical corticosteroids or antihistamines
TSH increase	All (30-60%)	Monitor TSH levels monthly and adjust thyroid-replacement medication dose
Hand-foot syndrome	Sorafenib (76%)	Prevention: local care of feet and hands, urea cream 10% on hands and feet, use cotton socks Treatment: Thick urea-based cream (30%), topical lidocaine Use comfortable shoes and avoid hot/cold water NSAIDs, codeine, or pregabalin
Alopecia	Sorafenib (67%)	Inform the patient that it is temporary, usually recovering after the treatment, and does not require any treatment
Proteinuria	Lenvatinib (31%)	If ≥ 2 g/24 hours: withhold treatment Resume at reduced dose when proteinuria is < 2 g/24 hours Discontinue if nephrotic syndrome
Mucositis	All (30%)	Mouthwash with lidocaine + sucralfate, salt and sodium bicarbonate, or chlorhexidine
Hypocalcemia	Sorafenib (18%)	Monitor blood calcium levels at least monthly and replace calcium + vitamin D as necessary
QTc prolongation	Vandetanib (23%, G > 3 in 14%)	Serially monitor ECG and electrolytes and correct any abnormality Avoid drugs known to prolong QTc Discontinue MKIs if QTc ≥ 500 msec

AEs: adverse events; ACEI: angiotensin converting enzyme inhibitor; ARB: angiotensin II receptor blockers; BB: beta-blockers; CCB: calcium channel blockers; NSAID: non steroidal anti-inflammatory drugs.

### When to stop an MKI treatment?

Therapy should be continued as long as the net benefit exceeds the net detriment ( 5 ). There is no general consensus, and the decision to withdraw any MKI is made on a case-by-case basis. These situations are listed in Table 4 ( 25 , 59 , 60 ).

**Table 4:** Factors that lead to MKI continuation or withdrawal

Scenario	Comment
Benefits of continuing treatment	Maintain stable disease or slow disease progression
Magnitude of tumor reduction	If after an initial significant tumor response, a slow disease progression occurs, treatment may be continued as long as the clinical benefit is maintained
Size and location of tumor foci	Evaluate risk of local complications if the tumor progresses
Feasibility of focal treatments	In patients with dissociated responses, treatment may be maintained in those who progress in a single or in a few metastases that may benefit from focal treatment modalities. This may occur with bone metastases that progress and may then benefit from focal treatment, whereas metastases in lungs, lymph nodes, or liver respond
Tolerance	AEs are significant and may lead to a dose reduction in 11-73% of patients and to MKI withdrawal in 7-25%. However, AEs can frequently be managed without the need for dose reduction or discontinuation of treatment. Also, the tolerance is highly variable from patient to patient and between different MKIs
Availability of other treatment modalities	Availability of other drugs or possibility to include patients in international protocols of new drugs

## CONCLUSION

RAI refractoriness is an uncommon situation, and many patients may survive in the absence of treatment for years or even decades with a stable or slowly progressive disease. However, a few patients may require treatment when the tumor burden is large and when progression has been documented. MKIs represent the first-line treatment for advanced refractory DTC: they significantly prolong PFS, and some induce a high objective response rate. However, at present there is no demonstrated benefit regarding overall survival, and the quality of life is altered during treatment. For these reasons, it is important to adequately select patients who should be treated and then manage them with an interdisciplinary approach.
